# ECG abnormalities among HIV infected children placed on ART at Enugu, South East of Nigeria

**DOI:** 10.4314/ahs.v20i4.26

**Published:** 2020-12

**Authors:** Emeka Spiff Eleazar, Clara Idara Eleazar, Daniel Chukwu Nwachukwu, Uchenna Ifeanyi Nwagha

**Affiliations:** 1 University of Nigeria, Department of Physiology; 2 University of Nigeria, Department of Microbiology; 3 University of Nigeria, Department of Physiology; 4 University of Nigeria, Department Physiology/Obstetrics & Gynaecology

**Keywords:** HIV, cardiovascular, children, HAART

## Abstract

**Background:**

Cardiovascular abnormalities are not much reported among human immunodeficiency virus (HIV) infected children especially in Africa where there is high HIV disease. In addition, the use of highly active antiretroviral therapy (HAART) in such children may have a protective effect on the cardiovascular system.

**Methods:**

Cross-sectional study of randomly selected eighty HIV infected and 80 aged matched non- HIV-infected children were used. HIV-infected children were on HAART for more than 5years and had steadily received the treatment for 6 months prior to the time of the tests. Heights and weights were measured and body mass index calculated. Cardiac indices evaluated were heart rate (HR), PR interval, QRS duration, QT/QTC Interval, P/QRS/T Axis, RV5/SV1 voltage and RV5+SV1 voltage.

**Results:**

The average heart rate was significantly higher among HIV infected children on HAART than their non-infected counterparts (P= 0.019). At 0.05 significance level, their PR interval was significantly higher than those in the control group (P=0.050). The average QRS duration result also showed a significant difference between that of test and control subjects (P = 0.022)

**Conclusion:**

The HAART usage possibly improved the cardiovascular functioning in the infected children but the protective effects diminish with increase age and longer exposure

## Introduction

The risk of cardiovascular diseases is increasingly recognized as serious major public health problem in individuals infected with HIV 1, 2. This problem may be as a result of long-term exposure to the virus, effects of ongoing inflammatory responses, progressive immunologic dysfunction, and/or long term adverse effects of ART. The major risk and prevalence of cardiovascular outcomes among HIV-infected children and adolescents is yet to be realized, though recent researches are exploring new cardiovascular health challenges that face children living with HIV 3. Some studies have shown the incidence and course of human immunodeficiency virus (HIV) infection in relation to cardiac illness in both children and adult 4. An approximate 8% to 10% of HIV patients have been reported to develop symptomatic heart failure over a 2- to 5-year period 5

Sub-clinical echocardiographic abnormalities can independently predict adverse outcomes of HIV infection as well identify high-risk groups to target for early intervention and therapy. However cardiovascular manifestations of HIV have been altered by the introduction and use of highly active antiretroviral therapy (HAART) regimens 4. Since the introduction of combination HAART, mortality, opportunistic and other related infections have significantly declined among children with perinatal HIV infection 6, 7. Children living with HIV are less likely to develop AIDS because of routine and early initiation of effective ART 8, 9.

Cardiovascular involvement is less well-reported in children, coupled with the chronic adverse effects of ART in the children living with HIV-infection 10. Although the cardiovascular effects of HIV and ART are not fully understood, HIV-infected children are routinely exposed to highly antiretroviral drugs (HAART) while the cardiovascular system is still developing. Sub-clinical cardiac abnormalities may develop into symptomatic cardiomyopathy in adulthood 11. Cardiac complications do give rise, significantly, to morbidity and mortality in HIV-infected children. There have been only few reports of cardiac manifestations in HIV-infected children in developing countries 12. Children offer unique opportunity to study the physiological mechanism of HIV-associated cardiomyopathy and pulmonary functions because they are less likely than adults to be exposed for long term to confounding factors as hypertension, smoking, obesity, diabetes mellitus and coronary atherosclerosis^13^, ^14^, ^15^. The study is aimed at assessing the cardiovascular functions of children of ages 7–18 years infected with HIV and placed on HAART.

## Methodology

### Study Design and Sampling

The study design was cross-sectional. The sampling was carried out between February 2016 and November 2017. The subjects were children attending HIV/AIDS clinic at University of Nigeria Teaching Hospital, Ituku, Ozalla and children recruited from Churches and other Children Organizations within Enugu metropolis. Eighty children aged 7–18 years living with HIV/AIDS were randomly selected and matched with 80 HIV controls. The HIV- infected children have been on HAART for more than 5years and had steadily received treatment for at least 6 months prior to the enrollment. Materials employed included Bio-data questionnaires. This provided the demographic data, family background effect, life style and socio economic data of the subjects. Ethical clearance was obtained from the Health Research and Ethics committee of the University of Nigeria Teaching Hospital Ituku-Ozalla in Enugu State. Informed Consent Forms (ICF) were given to the parents/guardians or legally accepted representatives (LAR) of the children.

### Inclusion and exclusion criteria

Inclusion criterion for subjects was HIV/AIDS positive children on HAART between 7–18 years of ages that were signed into the study by consent from their Legally Accepted Representatives (LAR). Also those that had received treatment and subsequently had undetectable viral load but were still on ART were included because of their previous exposure to the virus. Exclusion criteria were children with co-mobility such as sickle cell anaemia, scoliosis and known heart diseases or obstructive lung diseases.

Inclusions into the control group were children between 7–18 years of age that tested negative to HIV, and consenting to join the study by their LAR. Exclusion criteria were children with co-mobility such as sickle cell anemia, Scoliosis and known heart diseases or obstructive lung diseases.

### HIV testing and measurements of cardiac indices

HIV screening /testing kit (Alere HIV1/2 SET, REF 7D2343) was used to screen the control subjects. The test results were visually read, qualitative immunoassay for the detection of antibodies to HIV-1 and HIV-2. The test subjects were screened and confirmed HIV positives. Polymerase Chain Reaction (PCR) tests was also carried out at University of Nigerian Teaching Hospital Laboratory. Manual blood pressure equipment (mercury sphygmomanometer and stethoscope) was used for measuring blood pressures. Height and weight measuring equipment (Axiom Healthscale) were used in determining Body Mass Index. Electrocardiogram machine (M1200) was used in measurement and calculation of Heart Rate (HR), PR interval, QRS duration, QT/QTC Interval, P/QRS/T Axis, RV5/SV1 voltage, RV5+SV1 voltage.

### Data management and analysis

The average values were statistically, determined for each age group. Computer was used for recording, storage and analysis. Data analysis was carried out using SPSS version 16.0. Paired sample t-test for the various age group variables was determined. The probability values were determined using at 0.05 significance levels. P- Values less than 0.05 were regarded as significant.

## Results

**Figure 1:**
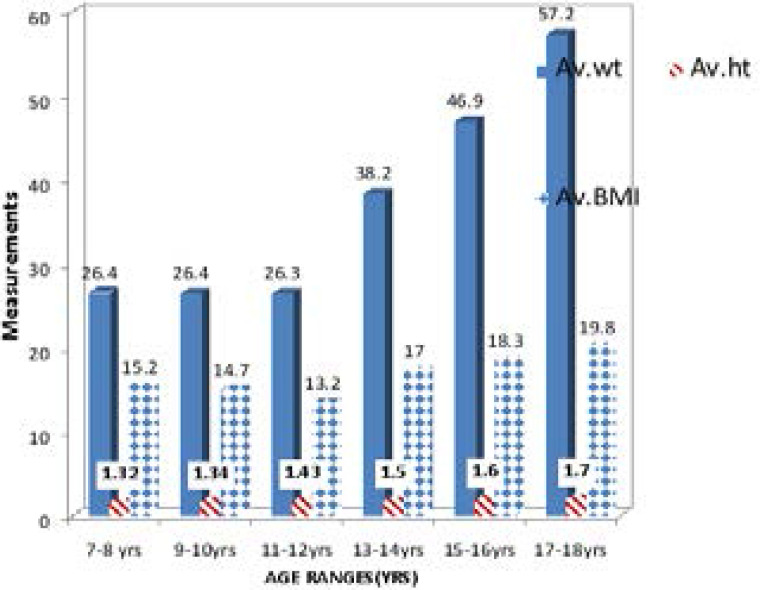
Height, Weight and BMI by Age Range AV. WT (Kg) =26.4 -57.2, AV. HT (meters) =1.32–1.70, AV BMI (Kg/m^2^) =15.2 -19.8

The measurements of the heights and weights of the HIV- infected children at the various age ranges from 7–18 years are shown in Figure 1. The average body mass index (Av. BMI) were 15.2, 14.7, 13.2, 17.0, 18.3 and 19.8, respectively. Table 1 displays the average BMI of the HIV-infected children and the non-infected (control) children comparatively. The Average (AV.) BMI (Kg/m2) for HIV infected and non-infected was significantly different (P=0.004).

**Table 1:** Average Body Mass Index(Kg/m^2^) by Age Ranges

Age ranges	Tests	Controls
7–8	15.2	16 .5
9–10	14.7	19.5
11–12	13.2	18.2
13–14	17.0	22.9
15–16	18.3	20.6
17–18	19.8	22.4

**Total Mean** 16.367 20.017

**Std. Deviation** 2.4533 2.4604

**Std. Error of Mean** 1.0016 1.0045

**Variance** 6.019 6.054

**P=0.004**

**Figure 2:**
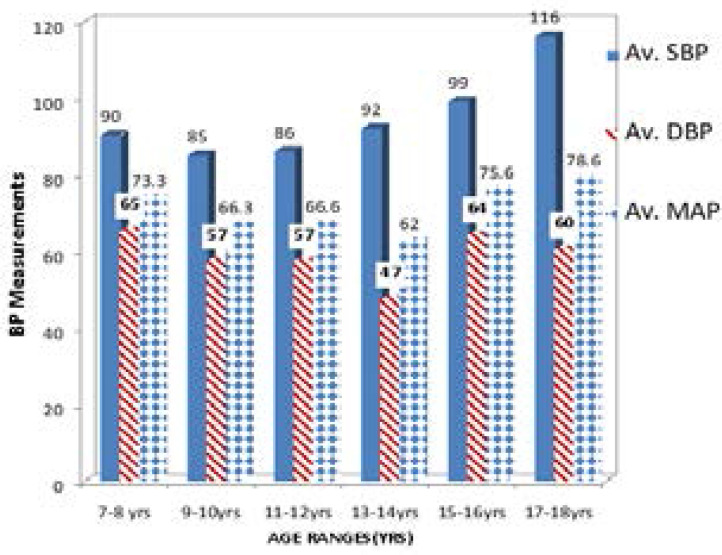
Blood pressure by age range • AV. Systolic Blood Pressure (SBP mmHg). • Av. Diastolic Blood Pressure (DBP mmHg). • Av. Mean Arterial Pressure (MAP mmHg).

The blood pressure measurements of the HIV-infected children are shown in Figure 2. The mean arterial pressure (MAP) for the various ages ranges were as follows: 7–8years (73.3), 9–10 years (66.3), 11–12 years (66.6), 13–14 years (62.0), 15–16 years (75.6) and 17–18 years (78.6). Table 2 displays the MAP of the test and control subjects. The MAP (mmHg) of test subjects was not significantly higher than that of the controls (P = 0.685).

**Table 2:** The Mean Arterial Pressure of Test and Control Subjects

Age ranges	Av. MAP -tests	Av. MAP-controls
7–8	73.3	65.1
9–10	66.3	66.4
11–12	66.6	73.0
13–14	62.0	74.6
15–16	75.6	70.9
17–18	78.6	80.3

**Total Mean** 70.400 71.717

**Std. Deviation** 6.3953 5.5919

**Std. Error of Mean** 2.6109 2.2829

**Variance** 40.900 31.270

P=0.685

The electrocardiograph (ECG) test results are recorded in table 3 and the comparison of the test and control data is displayed in Table 4. The average heart rate (Av. HR) of the HIV positive subjects was highest (98.0) at the age range of 7–8 years while the age range of 17–18 years recorded the lowest (73.0) average heart rate. Ages 9–10 years, 11–12 years, 13–14 years and 15–16 years recorded 89.3, 83.3, 83.1 and 81.4, respectively. The comparison of heart rate measurements of control and test subjects showed significant difference (P=0.050), at a p-value of < 0.05 significance level.

**Table 3:** Electrocardiography (ECG) Test Result by Age Range

Age Ranges	Av.HR(bpm)	Av. PR(MS)	Av. QRS(MS)	QT/QTC Interval (ms)	Av. P/QRS/ T Axis (ms)	Av. RV5/SV1 Voltage (Mv)	Av. RV5+SV1
7–8	98.0	143.13	72.9	342/438	53/54/50	1.40/0.94	2.34
9–10	89.3	156.80	80.2	357/436	46/49/50	1.10/1.04	2.14
11–12	83.3	168.23	78.8	346/405	43.3/60.5/53.8	1.50/1.30	2.80
13–14	83.1	158.16	75.6	352/413	37/57/36	1.50/1.03	2.53
15–16	81.4	181.80	76.6	360/416	48/40/39	1.60/0.90	2.50
17–18	73.0	180.50	85.8	376/408	49/44/46	2.0/0.9	2.90

**Table 4:** Electrocardiography (ECG) Result of Tests and Control Subjects by Age Ranges

Age	Av. HR. – Tests; Controls	Av. PR Interval- Tests; Controls	Av. QRS Duration(MS) Tests; Controls	Av. rv5+sv1 voltage(MV) Tests; controls
7–8	98 ; 85	143.13 ; 142.7	72.9 ; 77.7	2.34; 2.22
9–10	89.3 ; 84.7	156.80 ; 146.1	80.2 ; 89.7	2.14; 1.65
11–12	83.3 ; 83.6	168.23 ; 142.6	78.8 ; 93.4	2.80; 1.95
13–14	83.1 ; 75.7	158.16 ; 146.0	75.6 ; 86.7	2.53; 2.43
15–16	81.4 ; 77	181.80 ; 163.5	76.6 ; 92.5	2.50; 1.60
17–18	73.0 ; 63	180.50 ; 119.0	85.8 ; 124	2.90; 3.46
**P values**	0.019	0.050	0.022	0.001

For the average PR interval the highest reading was at the age range of 15–16 years which was 181.8, 7–8 years range recorded the lowest of 143.13. Average QRS duration of 85.8 was recorded for the age range of 17–18years followed by 80.2 recoded for the age range of 9–10 years. There was significant difference (P=0.022) in the Average QRS duration of the test and control subjects.

The QT/QTC interval for ages 7–18 years are displayed in table 3. The Av. RV5/SV1 were 1.40/0.94, 1.10/1.04, 1.50/1.30, 1.50/1.03, 1.60/0.90, 2.0/0.9 for the age ranges 7–18 years. The Av. RV5+SV1, appearing in descending order of the age ranges, were 2.34, 2.14, 2.80, 2.53, 2.50, and 2.90 (Table 3). There was significant difference (P=0.001) in the Av. RV5+SV1 of the test and control as shown in Table 4

## Discussion

The significant different in the BMI of the infected non-infected shows that HIV infection directly or indirectly affect the body mass index of the infected children. The increase heart rate in the children was possibly affected by ART treatment. The average Heart Rate (HR bpm) ranged from73.0- 98.0 for the infected and 63–85 for uninfected (P= 0.019). The HAART treatment could improve the heart rate however the protective effects of HAART exposure on cardiac function appeared to diminish eleven years after exposure^11^. Protease inhibitors (PI) has been shown to cause metabolic syndrome that may be associated with increased risk of cardiovascular disease^16^. Children infected with HIV by birth are routinely exposed to ART for years including in-utero, while the cardiovascular system is still developing. Fetal ART exposure may impair myocardial growth and improve depressed LV function 17. This means that the multi-agent HIV therapies that help sustain life may also directly increase the risk of cardiovascular events and accelerated atherosclerosis^18^, ^19^.

This research showed that average PR interval of test and control subjects was significantly different (P= 0.050). However, the PR interval of some of the HIV-infected children was above normal range. In infants and young children a PR interval ≥ 160 MS is long and also consistent with first-degree AV block 24. The QT interval depends on heart rate and age and has been shown to increase with age while decreasing with heart rate 20. On the contrarily, the result findings in this research did not show a progressively decreasing QT/QTC Interval. This study showed a significant difference in average QRS duration of infected (72.9–85.8) and uninfected (77.7–124.0) with P value of 0.022. The QRS duration represents the ventricular depolarization. The P wave duration and the QRS duration also increase with age. Prolongation of the QRS complex may be due to bundle branch block, ventricular hypertrophy, metabolic disturbances, or drugs 21. Children with HIV develop wild rage of cardiovascular problems from the sub clinical - electrocardiographic changes to the life threatening- cardiomyopathy; the exact causes are unknown, probably multi factorial 22. Lipshultz et al 23 also reported that cardiomyopathy is common in HIV-infected -children. In the dilated cardiomyopathy patients, mean 24-hour heart rate in beats per minute was significantly higher in comparison to controls 24. In Nigeria, cardiovascular dysfunction in HIV-infected children has been reported. A prevalence of 75.9% cardio vascular abnormalities was discovered to include pericarditis, myocarditis, dilated cardiomyopathy disease, pulmonary hypertension, cardiac failure, left ventricular systolic dysfunction, and increased left ventricular mass^25^. However, this study recorded no serious abnormalities showing that the children had not been on HAART treatment for prolonged period that would have caused the prolongation of the QRS complex

## Conclusion

Human immunodeficiency virus infections and AIDS clinical complications may affect the cardiovascular functioning in children during the development of cardiovascular system. But this study shows that early diagnosis of cardiac manifestation and prompt intervention by HAART did stop the progression of the complications. Study of the effect of the prolonged placement on HAART on adult patients within this region is recommended.
